# Evaluating the Medication Regimen Complexity Score as a Predictor of Clinical Outcomes in the Critically Ill

**DOI:** 10.3390/jcm11164705

**Published:** 2022-08-11

**Authors:** Mohammad A. Al-Mamun, Jacob Strock, Yushuf Sharker, Khaled Shawwa, Rebecca Schmidt, Douglas Slain, Ankit Sakhuja, Todd N. Brothers

**Affiliations:** 1 School of Pharmacy, University of West Virginia, 706 Health Sciences Ctr S, Morgantown, WV 26506, USA; 2 Graduate School of Oceanography, University of Rhode Island, 215 S Ferry Rd, Narragansett, RI 02882, USA; 3 Children’s National Hospital, 111 Michigan Ave. NW, Washington, DC 20010, USA; 4 School of Medicine, University of West Virginia, 1 Medical Center Dr, Morgantown, WV 26506, USA; 5 College of Pharmacy, University of Rhode Island, 7 Greenhouse Road Kingston, Narragansett, RI 02881, USA

**Keywords:** critical care, outcomes, patient safety, medication therapy management, electronic health records

## Abstract

Background: Medication Regimen Complexity (MRC) refers to the combination of medication classes, dosages, and frequencies. The objective of this study was to examine the relationship between the scores of different MRC tools and the clinical outcomes. Methods: We conducted a retrospective cohort study at Roger William Medical Center, Providence, Rhode Island, which included 317 adult patients admitted to the intensive care unit (ICU) between 1 February 2020 and 30 August 2020. MRC was assessed using the MRC Index (MRCI) and MRC for the Intensive Care Unit (MRC-ICU). A multivariable logistic regression model was used to identify associations among MRC scores, clinical outcomes, and a logistic classifier to predict clinical outcomes. Results: Higher MRC scores were associated with increased mortality, a longer ICU length of stay (LOS), and the need for mechanical ventilation (MV). MRC-ICU scores at 24 h were significantly (*p* < 0.001) associated with increased ICU mortality, LOS, and MV, with ORs of 1.12 (95% CI: 1.06–1.19), 1.17 (1.1–1.24), and 1.21 (1.14–1.29), respectively. Mortality prediction was similar using both scoring tools (AUC: 0.88 [0.75–0.97] vs. 0.88 [0.76–0.97]. The model with 15 medication classes outperformed others in predicting the ICU LOS and the need for MV with AUCs of 0.82 (0.71–0.93) and 0.87 (0.77–0.96), respectively. Conclusion: Our results demonstrated that both MRC scores were associated with poorer clinical outcomes. The incorporation of MRC scores in real-time therapeutic decision making can aid clinicians to prescribe safer alternatives.

## 1. Introduction

Medication Regimen Complexity (MRC) refers to multiple features of a patient’s medication drug regimen rather than an absolute number of medications consumed per day [1]. MRC incorporates features such as the number of agents, dosages, administration time intervals, and additional instructions (i.e., take on an empty stomach) [2,3,4]. An increase in MRC burden has been associated with poorer medication noncompliance and caregiver quality of life measures, as well as an increase in healthcare resource utilization [5,6,7,8,9]. Critical illness has been referred to as a subset of hospitalized individuals who are commonly afflicted with severe respiratory, cardiovascular, or neurological impairment, reflected in abnormal physiological observations. These patients are at a significant risk of higher MRC due to the severity of illness, the management of multiple chronic conditions, and the complex pharmacotherapies prescribed. It has been estimated that a critically ill adult may receive up to 13 medications per day and the chances of experiencing an adverse drug event are greater than 25% [10,11,12]. Therefore, examining only the quantity of medications administered may not accurately describe the complex and intricate nature of critical care medication therapy [13].

Numerous methods have been used to quantify the complexity of medication use. Yet, the most commonly utilized and validated objective scoring tool is the 65-item, weighted MRC Index (MRCI), which has been developed for outpatient use only [14,15,16,17,18]. The MRCI has been used to evaluate conditions such as neurological impairment in children and hypertension, diabetes, chronic obstructive pulmonary disease, and chronic kidney disease in adults [19,20,21,22,23,24]. The MRC for the intensive care unit (ICU) scoring tool has been developed and intended for use in critically ill patients [25,26]. MRC-ICU is the first validated quantitative weighted scoring tool intended to predict clinical events such as mortality [27,28]. A recent study demonstrates that the prediction of patient outcomes can be improved by incorporating the MRC-ICU score into the previously established severity-of-disease classification system Acute Physiology and Chronic Health Evaluation (APACHE II) scoring tool [29].

MRC scoring tools have the potential to aid in the identification of which patients may benefit from subsequent interventions (i.e., comprehensive medication review) [23,24]. In the critical care setting, the tool can further support the need for clinical pharmaceutical expertise and workload assignment to address the complex pharmacotherapeutic needs of the patient. However, the utilization of these tools has been limited due to the narrowly defined scope of the MRCI tool and the lack of substantial validation. To date, no studies have assessed the association between these two MRC scoring tools and clinical outcomes within the critical care setting.

In this study, we created two custom MRC scoring algorithms and several statistical and prediction models using the MRCI and MRC-ICU tools to investigate whether MRC scores are important predictors of clinical outcomes (i.e., ICU mortality, length of stay (LOS), and need for mechanical ventilation (MV)). We aimed to (1) examine how MRC scores correlate with clinical characteristics, (2) test the hypothesis that higher MRC scores are associated with poorer clinical outcomes, and (3) determine the utility of MRC scores as predictors of clinical outcomes.

## 2. Methods

### 2.1. Study Design and Setting

This was a single-center, STROBE-compliant retrospective cohort study of 322 patients enrolled into the ICU in a 220-bed community hospital in Providence, Rhode Island, USA, between 1 February 2020 and 30 August 2020. Due to the retrospective nature of the data, informed consent was not deemed necessary as all patient data were de-identified prior to use. The study was granted exemption by the Human Research Review Committee Roger Williams Medical Center (RWMC) Institutional Review Board (IRB: 00000058) and University of Rhode Island Institutional Review Board (IRB: 00000559). The data were curated and reviewed for accuracy by the RWMC data-extraction team.

### 2.2. Participants

All adults admitted to the ICU with general admission criteria were included in this study.

### 2.3. Inclusion and Exclusion Criteria

The patients were included if the following conditions were met: ≥18 years of age and admission to the ICU > 24 h. A total of 317 patients were included in the final analysis, five of whom were excluded due to extensive LOS (>40 days) and missing laboratory values.

### 2.4. Variables

Demographics, vital signs, laboratories, medication classes, and MV data were collected for each patient from the electronic health records (EHR) (Tables S1 and S2).

### 2.5. Data Sources

#### MRC Scoring Tools

MRC can be defined by the number of drugs and their dosing frequency. Two medication scoring indexes, the MRCI and MRC-ICU, have been developed for outpatient and critical care use, respectively. The total MRCI score is the weighted sum of 3 sections (dosage form, dosing frequency, and additional administration information), in which a higher score reflects a higher MRC burden. In computing the MRCI, both scheduled and ‘as needed’ medications and supplements are considered (see supplementary material). Despite the established use of the MRCI tool in the outpatient setting, we aimed to explore its utility in the ICU setting in this study [8].

Conversely, MRC-ICU is a 39-item, weighted, critically ill medication scoring tool comprising specific agents and classes (i.e., vancomycin—3 point; continuous intravenous saline—1 point) [26,30]. The MRC-ICU scoring calculation and individually assigned medication weights are provided in the supplementary material. The score has undergone validation testing for both internal and external validity [26,30]. Although an MRC-ICU score can be determined at any time during ICU admission, daily historical evaluation at 24 h intervals is most commonly utilized and was applied to our study. These two scores were calculated using custom codes for each patient at 24 and 48 h intervals.

### 2.6. Definitions

We defined cutoff values for both MRC scores based upon their distribution 24 h after ICU admission. We chose the median values as cutoff values, as there are no standardized cutoff values available for critically ill patients. The ‘high’ MRCI scoring cohort and the ‘high’ MRC-ICU cohort were defined as having cutoff scores of >63 and >6, respectively. Three clinical outcomes were measured: mortality, LOS, and need for MV for the ICU setting. We created a binary variable for LOS = 0 when LOS was < 48 h and LOS = 1 when LOS > 48 h. The need for MV was defined using a binary variable after 48 h of ICU admission (MV = 0, not mechanically ventilated; MV = 1, mechanically ventilated). Hemodynamic instability was defined as a hypotension (i.e., systolic blood pressure < 100 mmHg), mean arterial pressure < 65 mmHg, or an abnormal heart rate (i.e., arrhythmia or heart rate < 60 bpm or > 100 bpm).

### 2.7. Statistical Analysis

Descriptive statistics were used to describe the study population where continuous values were represented using means and interquartile ranges (IQRs). Categorical variables were described using frequencies and proportions. We conducted a descriptive analysis comparing survivor and non-survivor and low- and high-MRC-scoring groups using Student’s *t*, chi-squared (χ^2^), or Fisher’s exact tests to examine the relationships between clinical characteristics and the respective cohorts. Physiological and clinical characteristics were compared among the survivor and non-survivor cohorts. To account for the severity of illness, we included the following scoring tools: Simplified Acute Physiology Score (SAPS II), APACHE II, and Charlson comorbidity index (CCI). The MRCI and MRC-ICU were analyzed for mortality, need for MV, and SARS-CoV-2 (COVID-19) infection using the Wilcoxon signed-rank test. Four multivariable logistic regression models were utilized to identify the predictors of clinical outcomes (i.e., mortality, LOS, and need for MV) using severity scores, MRCI, MRC-ICU, and all the variables. The four models were: Model I—demographics, APACHE II, SAPS II, CCI, and 15 drug classes; Model II—demographics, MRCI_24 h, MRCI_48 h, CCI, and drug classes; Model III—demographics, MRC-ICU_24 h, MRC-ICU_48 h, CCI, and drug classes; and Model IV—all variables (see Table S3). In the LOS models, we excluded MRCI and MRC-ICU values at 48 h, as our threshold for binary values was 48 h after ICU admission. Significant predictors (*p* < 0.05) were selected for each model using a stepwise forward selection method. To further assess variable selection, we used an L1 penalization technique (LASSO). LASSO allows a more restrictive parameter selection to be performed that is minimally influenced by multicollinearity. The demographic variables included were age, sex, height, weight, body mass index (BMI), and race. Odds Ratios (OR) were calculated for each outcome of interest. All analyses were conducted using R, Version 4.0.0 (R Project for Statistical Computing), and the *glm*, *glmnet*, *and ggplot2 R* packages were used [31,32,33].

### 2.8. Prediction Model Development

To test the prediction ability of MRC scores for mortality, LOS, and need for MV, seven logistic classifier models were constructed without any variable selection (see Table S3). A ‘no imputation’ approach was used when preparing the data for the prediction model. We assessed correlated variables using the Pearson correlation coefficient. The SAPS II severity score was used in the prediction models due to a high correlation within the APACHE II classification system (Figure S1). The best fit models were selected using the best Akaike information criterion (AIC) measurement through an interactive process during cross-validation. We included all the predictor variables in each prediction model setup for exploring their individual role explicitly. Model performance was assessed via the area under the receiver operating characteristic (ROC) curve (AUC). An AUC of at least 0.7 was regarded as acceptable. We applied a ‘leave-one-out’ cross-validation method with 10,000 repetitions, and the AUC was selected as an overall performance measure. Additionally, sensitivity and specificity analyses were included for the three outcomes. Lastly, all prediction models recorded variable importance rankings for each clinical outcome.

## 3. Results

### 3.1. Demographic and Clinical Characteristics

Of the 317 patients included in the analysis, most were male (175 patients (55.2%)); and White (205 patients (65%)), with a median (interquartile range (IQR)) age of 62 (51–75) years. A total of 77% patients survived; 51% had an LOS > 48 h; and 31% required MV. Vital signs, serum electrolytes, and blood-cell values were similar among the survivor and non-survivor cohorts. Conversely, serum blood urea nitrogen (BUN) and creatinine values were significantly worse in the non-survivor cohort (25.7 mg/dL and 1.5 mg/dL vs. 38.7 mg/dL and 1.8 mg/dL), respectively. Non-survivors had a significantly longer duration on MV (147.2 h vs. 34.6 h) and a prolonged LOS (191.4 h vs. 87.4 h) than the survivor cohort. There was a high prevalence among both cohorts of acute respiratory failure with hypoxia (125 (39.4%)), COVID infection (52 (16.4%)), lactic acidosis (101 (31.9%)), kidney failure (96 (30.3%)), and acute myocardial infarction (78 (24.6%)) (Table 1).

### 3.2. Clinical Characteristics between MRC Cohorts

#### 3.2.1. Low- and High-MRC Cohorts

Among the higher-MRCI-scoring group, lower vital-sign values (systolic blood pressure, diastolic blood pressure, mean arterial pressure) were found to be significant (*p* < 0.01). Serum laboratory indices including phosphate, lactate, and albumin varied significantly among the MRCI cohorts. Higher MRCI scores were correlated with increased patient acuity when compared with lower-MRCI-scoring groups. Comorbidities such as hypo-osmolality, acute myocardial infarction, and unspecified sepsis were significant among both MRCI and MRC-ICU cohorts (Tables S4 and S5).

#### 3.2.2. Survivor and Non-Survivor Cohorts

**Figure 1:**
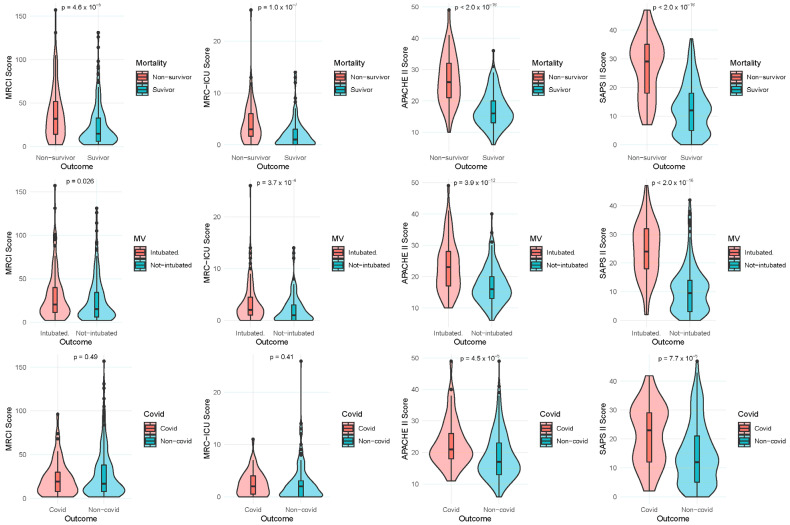
Comparisons among the severity score and MRC scores. Comparison of the severity-of-illness scores Acute Physiology and Chronic Health Evaluation (APACHE II), Simplified Acute Physiology Score (SAPS) II, Medication Regimen Complexity Index (MRCI), and Medication Regimen Complexity for the Intensive Care Unit (MRC-ICU) between survivors and non-survivors.

When compared with the non-survivor cohort, the survivor cohort had significantly lower APACHE II and SAPS II scores and a trend towards lower MRCI and MRC-ICU scores. In the COVID-19-infected cohort, APACHE II and SAPS II had significantly lower median values than in the non-COVID-infected group. Further, MRCI and MRC-ICU scores were significantly higher in the mechanically ventilated cohort (Figure 1). When analyzing age distribution by decade of life among different comorbidity severity indexes (i.e., Charlson, APACHE II, SAPS II) patients older than sixty years of age were associated with the highest severity index scores (Figure S2).

### 3.3. Medication Use

The top five medication classes prescribed among the high-scoring MRC cohorts (MRCI and MRC-ICU) were intravenous fluids (normal saline, 36% and 35%), gastrointestinal agents (pantoprazole, 29% and 32%), analgesics (acetaminophen, 26% and 26%), electrolytes (potassium chloride, 24% and 26%), and anti-infectives (piperacillin/tazobactam, 23%, and vancomycin, 27%). When incorporating the severity-of-illness scoring tools (APACHE II and SAPS II) with MRCI and MRC-ICU scores, patients with higher MRC scores (i.e., >63 MRCI and >6 MRC-ICU) were associated with increased mortality (14% and 15%), a longer LOS (i.e., >48 h; 30% and 34%), and an increased need for MV (24% and 28%), respectively (Figure S3).

### 3.4. Associations between MRC Scores and Clinical Outcomes

At admission, SAPS II was significant for all three outcomes: mortality (OR: 1.12 (1.07–1.18)), LOS (OR: 1.04 (1.0–1.11)), and need for MV (OR: 1.17 (1.13–1.21)) (Table 2), respectively. When only incorporating MRCI scores into the model (Model II), the MRCI score at 24 h was a significant predictor but showed slight associations with all outcomes with ORs of 1.01 (95% CI: 1.0–1.02), 1.01 (1.0–1.02), and 1.01 (1.01–1.02) for mortality, LOS, and need of MV, respectively. Further, MRCI scores at 48 h were found to be significant risk factors but weakly associated with mortality and need for MV (Table 2). MRC-ICU scores at 24 h (Model III) were significant risk factors in all outcomes in Model III with ORs of 1.12 (95% CI: 1.06–1.19), 1.17 (1.1–1.24), and 1.21 (1.14–1.29) for mortality, LOS, and need of MV, respectively. Notably, in Model III, Hispanic ethnicity was significantly (*p*-value < 0.001) associated with mortality 6.44 (3.45–12.41) and need for MV (2.21 (1.29–3.85) with ORs 6.44 (3.45–12.41) and 2.21 (1.29–3.85), respectively. Complimentary results from the LASSO model confirmed the risk-factor selection trends (Table S6). The use of vasopressors was found to be a significant risk factor for all clinical outcomes in Model IV. When evaluating morality, the use of paralytic agents was significant (OR: 3.38 (1.09–11.11)). The use of anti-infectives, anticoagulants, and cardiovascular agents was significantly associated with a prolonged LOS. Lastly, the use of analgesics, sedatives, psychiatric, cardiovascular, and pulmonary agents was a significant risk factor for the need of MV.

### 3.5. Role of MRC Scores in the Prediction of Clinical Outcomes

**Figure 2:**
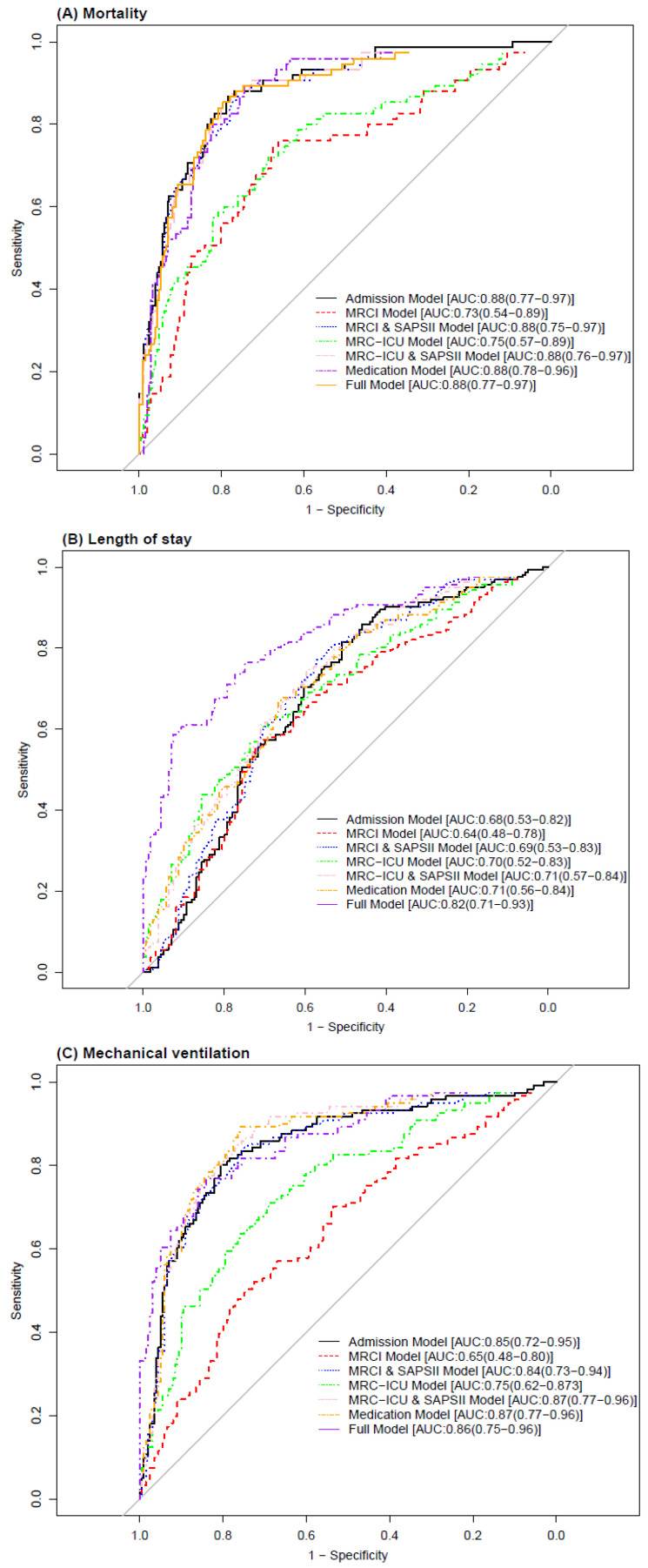
Performance of the prediction models for predicting the clinical outcomes. Receiver operating characteristic curves (AUCs) for (**A**) ICU mortality, (**B**) ICU length of stay, and (**C**) need for mechanical ventilation.

**Figure 3:**
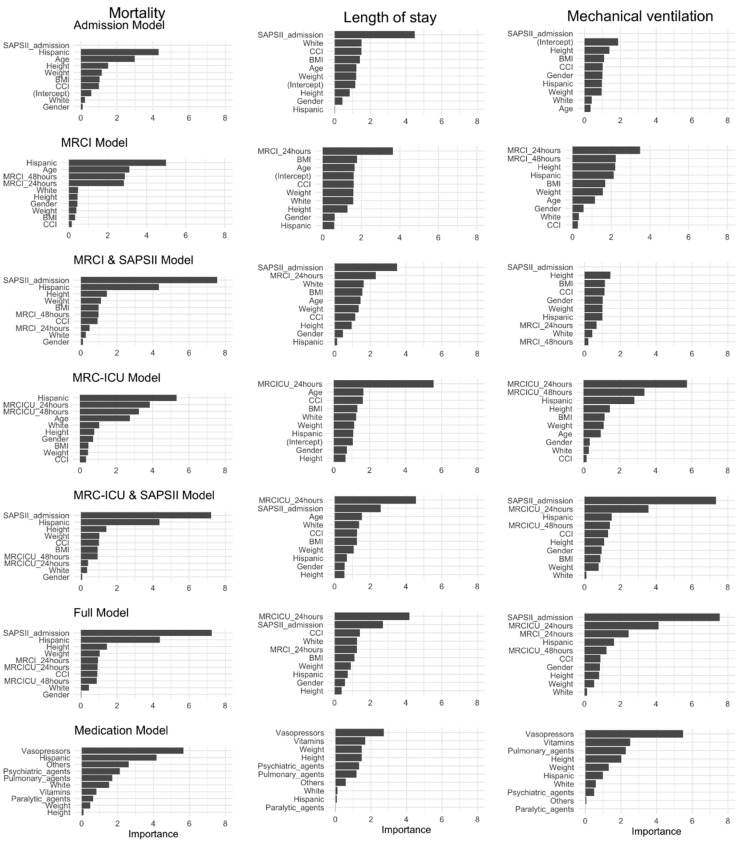
Variable importance of the prediction models. Top 10 variables of importance were ranked for 7 prediction models: ICU mortality (**left panel**), length of ICU stay (**middle panel**), and need for mechanical ventilation (**right panel**).

The Admission Model was found to be the best model (AUC: 0.88 (95% CI: 0.77–0.97)) to predict mortality (Table 3). However, models MRCI and SAPS II (AUC: 0.88 [0.75–0.97]) and MRC-ICU and SAPS (AUC: 0.88 [0.76–0.97]) performed similarly (Figure 2). In the MRCI and SAPS II Model, MRCI scores at 24 and 48 h were identified as the top variables of importance when predicting mortality (Figure 3). Further, vasopressors were the most important variable to predict mortality within the Medication Model. When predicting the LOS, the Medication Model (AUC: 0.82 [0.71–0.93]) outperformed all other models. Vasopressors and psychiatric agents were among the top five important variables to predict the LOS. Further, MRC scores at 24 h and 48 h were selected in the top 10 variable importance list for models including MRC scores (i.e., MRCI and SAPS II, and MRC-ICU and SAPS II).

When predicting the need for MV, MRC-ICU and SAPS II (AUC: 0.87 [0.77–0.96]) outperformed all other models. SAPS II and MRC-ICU at 24 and 48 h were among the top important variables to predict the need for MV. Lastly, vasopressors and pulmonary agents were among the top five medication classes identified when predicting the need for MV. Hispanic ethnicity was found to be one of the top important variables in the MRCI and MRC-ICU models for predicting mortality and need for MV (Figure 3).

## 4. Discussion

### 4.1. Clinical Characteristics between MRC and Survivor Cohorts

We found that both MRC scores varied widely within the cohort. The regression analysis confirmed that MRCI and MRC-ICU scores at 24 h were significantly associated with all outcomes: mortality, LOS, and need for MV. Secondly, higher MRC scores were associated with hemodynamic instability and higher APACHE II scores. Survivors had significantly lower MRCI, MRC-ICU, APACHE II, and SAPS II scores. Thirdly, the MRC-ICU and SAPS II Model improved the prediction of all three outcomes. Historically, the utility of MRC scores and their relationships with clinical outcomes in the critical care setting have not been fully established. Previous studies have suggested inconsistent findings when investigating the MRCI score with medication nonadherence and hospitalization in the outpatient settings [34,35,36]. In the ICU, MRC-ICU scoring has been correlated with mortality, but it has not been explored for the LOS nor the need for MV. This study explored the relationship between MRC-ICU scores and all three clinical outcomes. Although it did not meet statistical significance, our study suggested that non-survivors had poorer renal function, increased time on MV, and an extended ICU LOS [37,38,39]. Lastly, these findings suggest that MRC scores should be further investigated to determine their association with the LOS. Importantly, our findings have several real-world implications for the identification, clinical management, and potentially prevention of poorer clinical outcomes in critically ill adults with the highest MRC scores.

### 4.2. Associations between MRC Scores and Clinical Outcomes

Our results clearly demonstrated that both MRC scores at 24 h were associated with mortality, suggesting they could be considered for incorporation into current practice (Table 2). It is plausible to mention that MRC-ICU scores at 24 h showed slightly higher association with all three clinical outcomes than MRCI scores at 24 h. Moreover, the previously published ML model has demonstrated that MRC-ICU scores are associated with ICU mortality [29]. Historically, MRC has been shown to be a better risk factor of mortality than polypharmacy alone [35]. Interestingly, we found that Hispanic ethnicity was one of the top 10 important variables identified for predicting mortality, LOS, and need for MV. Historically, racial inequalities in critical illnesses and outcomes have been well described [40]. For example, African American patients have the highest disease burden requiring intensive care treatments and are more likely to die from sepsis. During the COVID-19 pandemic, Hispanic patients have been found to have higher ICU utilization and mortality than non-Hispanic patients [41].

### 4.3. Medication Use as Predictor of Clinical Outcomes

The use of vasopressors was a significant predictor in all clinical outcome models. In practice, the use of vasopressors is indicated in patients with poorer health conditions, such as decompensated heart failure and shock [42,43]. The frequent diagnosis of ICU-related delirium has been a known contributing factor to the ICU LOS among other undesirable outcomes [44,45,46]. Historically, numerous medications have been used to minimize the duration of delirium, yet studies to identify a safe and effective agent are lacking. Our findings of current psychiatric medications potentially contributing to an increase in time on MV suggest the continued need to identify an agent to minimize the incidence and duration of ICU delirium leading to extended time on MV and LOS.

The association of pulmonary and paralytic agent use with mortality and the need for MV was anticipated in our findings as these therapeutic classes are commonly associated with high-acuity diseases such as acute respiratory distress syndrome (ARDS) and acute brain injury [47,48]. Therefore, our study supports the inclusion of medication-class usage to predict critically ill outcomes. Most notably, the lack of medication-use evaluation into the existing severity-of-illness scoring tools (i.e., SAPS II and APACHE II) is a major shortcoming for their prediction accuracy. MRC scores may provide valuable information to bedside clinicians, including critical care pharmacists, who have been recognized as essential members of the interdisciplinary care team by major societal organizations [49,50].

### 4.4. Implications of These Findings

MRC scores can be calculated and incorporated into the EHR to readily identify patients at higher risk. During the COVID-19 pandemic, it has become even more evident that the healthcare system and, in particular, the access and utilization of critical care resources have become profoundly strained [51]. The early identification of higher-risk patients based upon MRC scores can aid in triaging limited ICU resources. Currently, there is no standardized MRC method for presenting safety alerts pertaining to medication use in the ICU. The development and integration of MRC scores into clinical decision support tools can alert interdisciplinary care team members to review and modify the medication regimen to ensure safer, patient-centered care [52,53,54,55]. This study corroborates the need of standardizing MRC scores within the critically ill population. The strength of our study findings is three-fold: (1) rigorous statistical investigation to identify the MRC score as a predictor of clinical outcomes, (2) evaluating the accuracy of incorporating MRC scores to predict clinical outcomes, and (3) investigating the utility and usability of MRC scores in the critically ill population.

Despite finding an association between MRC scores and all three clinical outcomes in our regression modeling exercise, neither score is without its unique limitations [6,56]. The MRCI has been established and validated for use in the outpatient setting only, but it does not incorporate influential critical care medications for ICU patients [7,8,18,56,57]. On the contrary, MRC-ICU does include critical care medications, yet it does not incorporate the complexities of the pharmacotherapeutic regimen, such as the medication combinations, dosages, or frequencies. Further, neither previously established scores consider pre-existing comorbidities or severity of critical illness, which are crucial when assessing critical care outcomes. Our results suggest that the inclusion of MRC scores into standardized severity index scoring tools (i.e., APACHE II, SAPS II) can improve the prediction of critical care outcomes (MRCI and SAPS II, and MRC-ICU and SAPS II models). However, these MRC scoring tools need to be further validated using a larger critically ill patient cohort to compare against existing standardized mortality prediction tools. We propose adopting MRC scores, pre-existing comorbidities, and severity of illness into future modeling to improve the accuracy of prediction.

### 4.5. Limitations

Our study must be considered in the context of several limitations. First, the retrospective nature of the study design exposes the risk of missing data that can contribute to confounding bias. For example, our findings suggest that psychiatric medications increase time on MV. However, these results can be confounded by (a) the exclusion of the weights in MRCI score calculation for important medications (i.e., vasopressors and anti-infectives), (b) the exclusion of weights in MRC-ICU score calculation for over-the-counter medications, (c) the exclusion of patient’s disease severity, and (d) the exclusion of multiple combinations of regimens. Second, we were unable to measure the previous exposure of MRC prior to ICU admission due to unconfirmed and varying pre-admission medication use. Third, these results may have been subjected to residual biases and unmeasured confounders due to the exclusion of commonly associated conditions (i.e., diabetes, hypertension, and dyslipidemia) as contributing factors to MRC scores. Fourth, selection biases could have occurred as general admission criteria applied to patient selection regardless of disease severity and prior to healthcare resource utilization. The generalizability of these findings is limited as they may not apply to patients with specific and life-threatening diseases such as ARDS, decompensated heart failure, and sepsis. Lastly, both MRC scores at 24 and 48 h can be indirectly related to the LOS, as a higher LOS may constitute higher MRC scores for the patients who remained admitted to the ICU for more than 48 h. Further research is necessary to control for the identified biases through a multi-centered randomized controlled trial among critically ill patients.

## 5. Conclusions

In this retrospective cohort study, our findings suggested that higher MRC scores were associated with poorer clinical outcomes (i.e., ICU mortality, LOS, and need for MV). Moreover, we found that MRC scores in conjunction with current severity-of-illness scores (i.e., APACHE II and SAPS II) improved the accuracy of the prediction of clinical outcomes. However, the future application of these findings needs to be validated using large EHR datasets from a more diverse patient population. Lastly, adopting these tools into the daily clinical practice could become the standard of care.

## Figures and Tables

**Table 1:** Demographic and clinical characteristics between survivor and non-survivor cohorts.

Characteristics	Survivors (*n* = 243)	Non-Survivors (*n* = 74)	*p*-Value
Demographics			
Age, median (IQR), y	60.8 (48–73.5)	65.8 (58–76)	0.81
Sex, No. (%)			
Male	134 (55)	41 (55)	>0.99
Race, No. (%)			
White	163 (67)	42 (57)	0.14
Black	19 (8)	7 (9)	0.83
Hispanic	25 (10)	12 (16)	0.24
Asian	4 (2)	0 (0)	0.61
BMI, median (IQR)	29 (23–32)	28.5 (23–31.8)	0.44
Vital Signs			
Systolic blood pressure (mm Hg)	122.8 (108–133.7)	107.1 (96.1–118.4)	0.22
Diastolic blood pressure (mm Hg)	71.6 (62.3–80.4)	64.2 (55.4–71.6)	0.38
Mean arterial pressure (mm Hg)	88.7 (77.7–97.5)	78.5 (69.4–86.4)	0.22
Heart rate (beats/min)	88.7 (78–99.1)	99.4 (86.2–113.5)	0.82
Respiratory rate (breaths/min)	19.2 (16–21)	25.2 (21.4–28)	0.09
Temperature (°C)	98.2 (97.7–98.7)	97.8 (96.8–99.2)	0.74
SaO_2_ (mm Hg)	96.7 (95.1–98.7)	93.9 (92.5–97)	0.77
Serum Laboratory Values			
Sodium (mEq/L)	136.3 (134–139)	138.1 (134.6–143)	0.58
Potassium (mEq/L)	4 (3.6–4.4)	4.4 (3.9–4.9)	0.16
Chloride (mg/dL)	103.9 (101–108)	102.7 (98–108.5)	0.35
Carbon dioxide (mEq/L)	23.6 (21–26)	20.7 (14.2–25.9)	0.96
BUN (mg/dL)	25.7 (12–30)	38.7 (21.2–50)	0.03
SCr (mg/dL)	1.5 (0.7–1.4)	1.8 (0.9–2.5)	0.05
Glucose (mg/dL)	168.5 (110.5–198)	219.5 (124–251.2)	0.30
Calcium (mg/dL)	8.2 (7.7–8.7)	8.1 (7.2–8.5)	0.21
Magnesium (mg/dL)	1.9 (1.6–2.1)	2.3 (1.9–2.5)	0.12
Phosphate (mg/dL)	3.5 (2.8–4)	6.1 (3.8–7.6)	0.26
White blood cell (×10^3^/mL)	11 (7.2–13.8)	15.6 (8.4–19.4)	0.51
Hemoglobin (g/dL)	10.2 (8.6–11.8)	9.7 (8.1–11.6)	0.31
Hematocrit (%)	31.8 (27.6–36.2)	31.4 (24.9–36)	0.35
Platelets (×10^3^/mL)	211.3 (143–268)	215.5 (128.5–271.8)	0.57
Lactate (U/L)	3 (1.4–3.3)	8.2 (3.1–13.5)	0.41
PT (s)	17.6 (11.5–16.1)	19.9 (13.3–27.8)	0.56
INR	1.7 (1.1–1.6)	2 (1.3–2.8)	0.56
Albumin (g/L)	3.1 (2.8–3.5)	2.8 (2.3–3.2)	0.71
Total bilirubin (mg/dL)	1.3 (0.5–1.2)	2.5 (0.5–1.2)	0.87
Urine Output every 6 h (mL/h)	60.4 (7–96.5)	56.5 (10.3–58.4)	0.49
eGFR (mL/min/1.73 m^2^)	71.2 (42–99.2)	50.3 (26.8–70.5)	0.11
Durations			
Time on mechanical ventilation (h)	34.6 (0–0)	147.2 (4–197.5)	0.08
ICU length of stay (h)	87.4 (21–86)	191.4 (22–282.5)	0.08
Scoring Assessment on ICU admission			
APACHE II	17 (13–20)	27.1 (21–32)	0.01
SAPS II	12.5 (5–18)	27.5 (18.2–35)	0.039
MRCI	62.9 (34–84)	81.1 (43–104.5)	0.339
MRC-ICU	6 (3–8)	8.6 (4–12)	0.107
GCS at admission	13.1 (12–15)	7.2 (3–11)	0.351
Lactic acidosis (E87.2)	62 (26)	39 (53)	<0.001
Hypokalemia (E87.6)	83 (34)	19 (26)	0.22
Kidney failure (N17.9)	63 (26)	33 (45)	0.004
Hypo-osmolality hyponatremia (E87.1)	63 (26)	26 (35)	0.16
Was not resuscitated (Z66)	35 (14)	51 (69)	<0.001
Acute myocardial infarction (I21.A)	52 (21)	26 (35)	0.025
Unspecified sepsis (A41.9)	53 (22)	23 (31)	0.14

BUN, blood urea nitrogen; Scr, serum creatinine; eGFR, estimated glomerular filtration rate; PT, prothrombin time; APACHE II, Acute Physiology and Chronic Health Evaluation II; SAPS II, Simplified Acute Physiology Score II; MRCI, Medication Regimen Complexity Index; MRC-ICU, Medication Regimen Complexity in the Intensive Care Unit.

**Table 2:** Four logistic regression models for three clinical outcomes: mortality, length of ICU stay (LOS), and need for mechanical ventilation (MV). List of selected variables using stepwise selection method for the four logistic regression models and their associations with mortality, LOS, and need for MV.

Selected Features	Mortality OR (95% CI)	*p*-Value	Length of ICU Stay OR (95% CI)	*p*-Value	Mechanical Ventilation OR (95% CI)	*p*-Value
Model I						
Age	1.02 (1.0–1.05)	0.12	1.02 (1.0–1.03)	0.05	-	-
Body mass index (BMI)	-	-	1.02 (1.0–1.05)	0.11	-	
White	-	-	0.59 (0.35–0.98)	-	-	-
Hispanic	6.11 (2.84–13.85)	<0.001	-	-	-	-
SAPS II at admission	1.12 (1.07–1.18)	0.001	1.04 (1–1.11)	<0.001	1.17 (1.13–1.21)	<0.001
APACHE II at admission	1.13 (1.05–1.21)	0.002	0.92 (0.88–0.98)	0.15	-	-
CCI	-	-	1.13 (1.0–1.32)	0.14	-	-
Model II						
Age	1.03 (1.02–1.05)	<0.001	1.01 (1.0–1.03)	0.09	-	-
Height	-	-	1.14 (1.01–1.35)	0.11	1.02 (1.04–1.46)	0.03
Weight	-	-	0.95 (0.89–1.01)	0.09	0.95 (0.89–1.01)	0.1
Body mass index (BMI)	-	-	1.17 (1.0–1.41)	0.06	1.18 (1.0–1.42)	0.07
White	-	-	0.56 (0.33–0.93)	0.03	-	-
Hispanic	5.74 (3.15- 10.79)	<0.001	-	-	1.84 (1.11–3.07)	0.02
MRCI score at 24 h	1.01 (1–1.02)	0.003	1.01 (1.0–1.02)	<0.001	1.01 (1.01–1.02)	<0.001
MRCI score at 48 h	1.01 (1–1.02)	0.004	-		1.01 (1.0–1.02)	0.03
CCI	-	-	1.13 (1.0–1.32)	0.13	-	-
Model III						
Age	1.03 (1.02–1.05)	<0.001	1.01 (1.0–1.03)	0.1	-	-
White	-	-	0.59 (0.35–0.99)	0.04	-	-
Hispanic	6.44 (3.45–12.41)	<0.001	-	-	2.21 (1.29–3.85)	0.004
MRC-ICU at 24 h	1.12 (1.06–1.19)	<0.001	1.17 (1.1–1.24)	<0.001	1.21 (1.14–1.29)	<0.001
MRC-ICU at 48 h	1.1 (1.04–1.17)	0.002	1.05 (1.0–1.11)	0.7	1.11 (1.05–1.18)	0.001
CCI	-	-	1.14 (1.0–1.34)	0.1	0.84 (0.65–1.07)	0.155
Model IV						
Age	1.04 (1.01- 1.08)	0.017	-	-	-	-
Hispanic	6.23 (2.55- 16.41)	0.001	-	-	-	-
SAPS II at admission	1.09 (1.04–1.15)	0.001	1.04 (1–1.08)	0.072	1.17 (1.11–1.23)	<0.001
APACHE II at admission	1.14 (1.05–1.23)	0.002	0.94 (0.89–1)	0.038	-	-
MRCI at 24 h	-	-	0.99 (0.98–1)	0.134	0.97 (0.95–0.99)	0.001
MRC-ICU at 24 h	-	-	1.1 (1–1.22)	0.05	1.3 (1.13–1.51)	<0.001
CCI	0.79 (0.6–1.03)	0.093	-	-	0.77 (0.58–1)	0.052
Anti-infectives	-	-	2.27 (1.15–4.57)	0.019	-	-
Anticoagulants	0.38 (0.1–1.42)	0.139	2.26 (1.02–5.29)	0.05	-	-
Psychiatric agents	-	-	1.8 (0.96–3.36)	0.065	2.52 (1.01–6.64)	0.05
Pulmonary agents	-	-	-	-	3.14 (1.34–7.66)	0.01
Cardiovascular agents	-	-	2.81 (1.46–5.53)	0.002	0.4 (0.15–0.98)	0.05
Diuretics	-	-	3.35 (1.74–6.62)	<0.001	-	-
Analgesics sedatives	-	-	-	-	6.96 (1.73–36.07)	0.012
Vasopressors	5.55 (2.12–15.26)	0.001	3.49 (1.63–7.75)	0.002	5.75 (2.4–14.48)	<0.001
Paralytic agents	3.38 (1.09–11.11)	0.039	-	-	-	-
Vitamins	-	-	1.63 (0.88–3.03)	0.122	0.25 (0.09–0.62)	0.004
Others	2.54 (1.08–6.15)	0.034	-	-	-	-

The final models of logistic regression are reported using Odds Ratios (ORs) and 95% confidence intervals of risk factors for logistic regression. If the variable was not selected, the cell was marked with ‘-’. Bold ORs for logistic regression were significant. Model I: Demographics, Acute Physiology and Chronic Health Evaluation (APACHE II), Simplified Acute Physiology Score (SAPS II), Charlson comorbidity index (CCI), and drug classes. Model II: Demographics, Medication Regimen Complexity Index (MRCI_24 h), MRCI_48 h, CCI, and drug classes. Model III: Demographics, Medication Regimen Complexity in Intensive Care Unit (MRC-ICU_24 h), MRC-ICU_48 h, CCI, and drug classes. Model IV: all variables.

**Table 3:** Comparison of the prediction models. Prediction evaluation for ICU mortality, LOS, and need for mechanical ventilation. (The best 3 prediction results are noted in bold font, and demographic variables are included in each of the models).

	AIC	AUC	Sensitivity	Specificity
ICU Mortality				
Admission Model	222.007 (217.45–222.15)	0.88 (0.77–0.97)	0.72 (0.60–0.82)	0.89 (0.85–0.92)
MRCI Model	313 (308–313)	0.73 (0.54–0.89)	0.73 (0.60–0.84)	0.89 (0.85–0.93)
MRCI and SAPS II Model	225 (220–225)	0.88 (0.75–0.97)	0.73 (0.63–0.83)	0.89 (0.84–0.93)
MRC-ICU Model	302 (297–302)	0.75 (0.57–0.89)	0.73 (0.62–0.83)	0.89 (0.85–0.92)
MRC-ICU and SAPS II Model	225 (221–225)	0.88 (0.76–0.97)	0.73 (0.62–0.84)	0.90 (0.85–0.94)
Medication Model	236 (232–236)	0.88 (0.78–0.96)	0.61 (0.48–0.73)	0.86 (0.82–0.90)
Full Model	226 (221–226)	0.88 (0.77–0.97)	0.73 (0.62–0.84)	0.89 (0.85–0.92)
Length of ICU Stay				
Admission Model	422.74 (421–423)	0.68 (0.53–0.82)	0.63 (0.5–0.70)	0.62 (0.54–0.70)
MRCI Model	431 (429–431)	0.64 (0.48–0.78)	0.65 (0.58–0.71)	0.62 (0.55–0.71)
MRCI and SAPS II Model	421 (419–422)	0.69 (0.53–0.83)	0.64 (0.57–0.70)	0.62 (0.55–0.69)
MRC-ICU Model	404 (402–405)	0.70 (0.52–0.83)	0.64 (0.56–0.74)	0.61 (0.55–0.70)
MRC-ICU and SAPS II Model	401 (399–402)	0.71 (0.57–0.84)	0.63 (0.57–0.70)	0.62 (0.56–0.70)
Medication Model	323 (320–324)	0.82 (0.71–0.93)	0.75 (0.69–0.81)	0.74 (0.68–0.80)
Full Model	402 (399–403)	0.71 (0.56–0.84)	0.64 (0.58–0.71)	0.62 (0.54–0.70)
Need for Mechanical Ventilation				
Admission Model	304.62 (300.23–305.0)	0.85 (0.72–0.95)	0.79 (0.70–0.86)	0.8 (0.75–0.85)
MRCI Model	408 (406–409)	0.65 (0.48–0.80)	0.79(0.69–0.86)	0.80 (0.75–0.85)
MRCI and SAPS II Model	308 (304–308)	0.84 (0.73–0.94)	0.77 (0.69–0.86)	0.80 (0.75–0.85)
MRC-ICU Model	365 (362–366)	0.75 (0.62–0.873)	0.78 (0.69–0.86)	0.80 (0.76–0.84)
MRC-ICU and SAPS II Model	290 (286–291)	0.87 (0.77–0.96)	0.78 (0.71–0.86)	0.80 (0.75–0.86)
Medication Model	273 (269–274)	0.86 (0.75–0.96)	0.8 (0.71–0.88)	0.81 (0.76–0.86)
Full Model	286 (281–286)	0.87 (0.77–0.96)	0.78 (0.70–0.86)	0.80 (0.76–0.85)

Abbreviations: Akaike information criterion (AIC), receiver operating characteristic curve (AUC). AUC is presented as a median value and 95% CI.

## Data Availability

The data supporting the findings from the study are available from the corresponding author upon a reasonable request based on the approval by the medical institution.

## Supplementary Materials

The following supporting information can be downloaded at: www.mdpi.com/article/10.3390/jcm11164705/s1, Table S1: Variables extracted from electronic health record data. Description of the variables extracted by group: demographics, vital signs, laboratories, scoring tools, medication classes, and comorbidities, Table S2: Grouping of medication classes according to their pharmacologic classification, Table S3: Model specifications of logistic regression model and prediction models for intensive-care-unit mortality, length of stay, and mechanical ventilation, Table S4: Comparison between patient characteristics and Medication Regimen Complexity Index (MRCI) scores. Demographic and clinical characteristics between low- and high-MRC-scoring cohorts, Table S5: Comparison between patient characteristics and Medication Regimen Complexity Intensive Care Unit (MRC-ICU) score. Demographic and clinical characteristics between low-and high-MRC-ICU-score cohorts, Table S6: Alternative method for variable selection. Four L1 penalization techniques (LASSO) for ICU mortality, ICU length of stay, and need for mechanical ventilation, Figure S1: Correlation among the severity and medication regimen scores. Pearson correlation Coefficients for severity of illness scores stratified by age groups: Charlson comorbidity index (CCI), Acute Physiology and Chronic Health Evaluation (APACHE II), Simplified Acute Physiology Score (SAPS) II, Medication Regimen Complexity Index (MRCI), and Medication Regimen Complexity in Intensive Care Unit (MRC-ICU). *p*-values should be interpreted as *: *p*-value < 0.05, **: *p*-value < 0.01, and ***: *p*-value < 0.001, Figure S2: Distribution of the severity and medication regimen scores. Distribution of severity-of-illness scores stratified by age groups: Charlson comorbidity index (CCI), Acute Physiology and Chronic Health Evaluation (APACHE II), Simplified Acute Physiology Score (SAPS) II, Medication Regimen Complexity Index (MRCI), and Medication Regimen Complexity in Intensive Care Unit (MRC-ICU), Figure S3: Bivariate analysis of severity-of-illness scoring tools (APACHE II and SAPS II) with MRC scores (MRCI and MRC-ICU). Abbreviations: Acute Physiology and Chronic Health Evaluation (APACHE II), Simplified Acute Physiology Score (SAPS) II, Medication Regimen Complexity Index (MRCI), and Medication Regimen Complexity in Intensive Care Unit (MRC-ICU). References [1,29,30] are cited in the supplementary materials.
