# Supplementary material for: Evaluating the Medication Regimen Complexity Score as a Predictor of Clinical Outcomes in the Critically Ill
Source: J Clin Med. 2022 Aug 11;11(16):4705. doi: 10.3390/jcm11164705 (PMC9410153; doi:10.3390/jcm11164705)
Supplement: Supplementary file 1 [file jcm-11-04705-s001.zip › Supp FigS3.pdf]

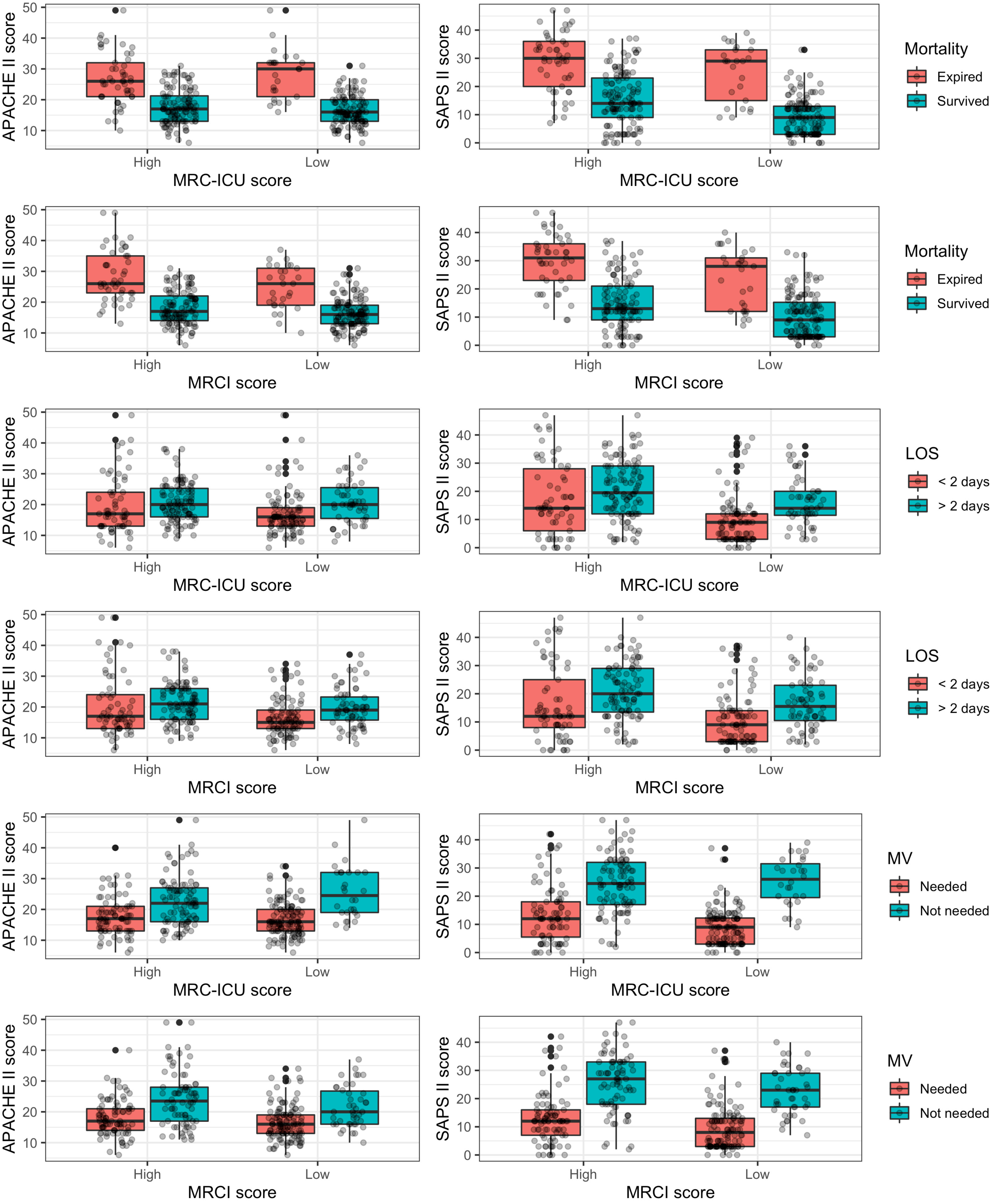

APACHE II score
SAPS II score
MRC-ICU score
MRCI score
High
Low
Mortality
Expired
Survived
LOS
< 2 days
> 2 days
MV
Needed
Not needed
