# Supplementary material for: Evaluating the Medication Regimen Complexity Score as a Predictor of Clinical Outcomes in the Critically Ill
Source: J Clin Med. 2022 Aug 11;11(16):4705. doi: 10.3390/jcm11164705 (PMC9410153; doi:10.3390/jcm11164705)
Supplement: Supplementary file 1 [file jcm-11-04705-s001.zip › Supplementary file.pdf]

Medication Regimen Complexity Index (MRCI)

Medication regimen complexity was computed using the MRCI tool (George et al., 2004). The validated tool consisted of three sections: dosage forms, dosing frequencies, and additional directions. MRCI scores were calculated based on pre-assigned weights for these elements. In which, a higher MRCI score indicates a more complex medication regimen. Table (1) describes the corresponding weights for each section of the tool. In cases where there is no matching option, the user can chose the closest section (e.g., six times daily, referred to as, 'q4h'). The details of each section can be found in the publication by George et al, 2004, Table A (dosage forms), Table B (dosing frequency), and Table C (additional directions).

Medication Regimen Complexity in Intensive Care Unit (MRC-ICU)

The MRC-ICU score consists of 39 components of different medication therapies (e.g., vancomycin, aminoglycosides, heparin) and includes a weighted scoring system for the relative complexity of each individual agent (e.g., vancomycin weighted as 3 points, whereas a continuous infusion of crystalloid fluids is weighted as 1 point) [2]. For an example, if a patient is receiving norepinephrine, vancomycin, IV fluid, argatroban, lithium, and a fentanyl infusion, the corresponding MRC-ICU score would be calculated as [1+3+1+2+3+2 = 12]. The MRC-ICU scoring weights and list of medications are provided elsewhere (https://journals.sagepub.com/doi/suppl/10.1177/1060028020959042) [3].

[1] George J, Phun YT, Bailey MJ, Kong DC, Stewart K. Development and validation of the medication regimen complexity index. Ann Pharmacother. 2004;38(9):1369-1376. doi:10.1345/aph.1D479

[29] Al-Mamun, M. A., Brothers, T., & Newsome, A. S. (2021). Development of machine learning models to validate a medication regimen complexity scoring tool for critically ill patients. Annals of Pharmacotherapy, 55(4), 421-429.

[30] Newsome, AS, Smith, SE, Olney, WJ, et al. Medication regimen complexity is associated with pharmacist interventions and drug-drug interactions: A use of the novel MRC-ICU scoring tool. J Am Coll Clin Pharm. 2020; 3: 47– 56. https://doi.org/10.1002/jac5.1146.
