# Supplementary material for: Evaluating the Medication Regimen Complexity Score as a Predictor of Clinical Outcomes in the Critically Ill
Source: J Clin Med. 2022 Aug 11;11(16):4705. doi: 10.3390/jcm11164705 (PMC9410153; doi:10.3390/jcm11164705)
Supplement: Supplementary file 1 [file jcm-11-04705-s001.zip › Table S1.pdf]

Table S1. Variables extracted from electronic health record data. Description of the variables extracted by group: demographics, vital signs, laboratories, scoring tools, medication classes, and comorbidities.

Demographics	Vital signs	Laboratories	Scoring tools	Medication classes	Comorbidities ICD-10 codes
Age (years)	SBP (systolic blood pressure)	Na (serum sodium level)	eGFR (estimated glomerular filtration rate)	IV fluid/electrolyte/total parenteral nutrition	E87.5 (hyperkalemia)
Sex (male) [n%]	DBP (diastolic blood pressure)	K (serum potassium level)	GCS (Glasgow coma scale)	Anti-infectives	E87.6 (hypokalemia)
BMI (kg/m^2)	MAP (mean arterial pressure)	Cl (serum chloride level)	APACHE II score (acute physiologic assessment and chronic health evaluation)	Analgesics and Sedatives	E83.42 (hypomagnesemia)
Ethnicity [n%]	HR (heart rate)	CO_2 (serum bicarbonate level)	SOFA score (sequential organ failure assessment score)	Cardiovascular	E87.1 (hypo-osmolality + hyponatremia
White	RR (respiratory rate)	BUN (blood urea nitrogen)		Pulmonary	I10 (hypertension)
African Black	Temperature	SCr (serum creatinine level)		Hematologic/ anticoagulants	J96.01 (acute respiratory failure with hypoxia)

Other		Glucose (serum glucose level)		Gastrointestinal	N17.9 (acute kidney failure)
		Ca (serum calcium level)		Vasopressors	I21.A1 (myocardial infarction)
		Mg (serum magnesium level)		Paralytics	A41.9 (sepsis)
		PO_4 (serum phosphate level)		Psychiatric	
		WBC (white blood cell)		Endocrine	
		Hgb (hemoglobin)		Diuretics	
		Hct (hematocrit)		Genitourinary	
		Platelet		Vitamins/ Supplements	
		Lactate (serum lactate level)		Other	
		PT (prothrombin time)			
		INR (international			

		normalized ratio)			
		Albumin			
		Total bilirubin			
		Arterial Blood Gas			
		pH			
		$PaCO_2$ (partial pressure of carbon dioxide)			
		PaO_2 (partial pressure of oxygen)			
		HCO_3 (bicarbonate)			
