# Supplementary material for: Evaluating the Medication Regimen Complexity Score as a Predictor of Clinical Outcomes in the Critically Ill
Source: J Clin Med. 2022 Aug 11;11(16):4705. doi: 10.3390/jcm11164705 (PMC9410153; doi:10.3390/jcm11164705)
Supplement: Supplementary file 1 [file jcm-11-04705-s001.zip › Table S2.pdf]

Table S2. Grouping of medication classes according to their pharmacologic classification.

Category	Definitions	Sample medications
IV fluid/electrolyte/total parenteral nutrition	Intravenous fluids, serum electrolyte replacements, and intravenous nutritional agents	Dextrose 5% in water, potassium chloride, amino acids
Anti-infectives	Antibiotics, antifungal, antiviral medications	Vancomycin, fluconazole, acyclovir
Analgesics and Sedatives	Systemic pain-relieving agents and sedating agents	Morphine, fentanyl, lorazepam, phenobarbital
Cardiovascular	Blood pressure lowering and anti-arrhythmic agents	Lisinopril, metoprolol, digoxin
Pulmonary	Inhaled bronchodilators, anti-inflammatory agents	Albuterol, ipratropium, budesonide
Hematologic/anticoagulants	Blood thinners	Heparin, enoxaparin, apixiban
Gastrointestinal	Anti-histamines, proton pump inhibitors, laxatives	Famotidine, pantoprazole, colace, senna, octreotide
Vasopressors	Blood pressure increasing agents	Norepinephrine, dopamine
Paralytics	Skeletal muscle paralytic agents	Cisatracurium, vecuronium
Psychiatric	Anti-schizophrenic, anti-depressants, anti-dopaminergic agents	Olanzapine, sertraline, haloperidol, gabapentin
Endocrine	Blood sugar lowering agents, thyroid supplementation, systemic steroids	Insulin, levothyroxine, prednisone
Diuretics	Agents to stimulate urine production and elimination	Furosemide, bumetanide
Genitourinary	Bladder anti-spasmodic, prostate medications	Oxybutynin, tamsulosin
Vitamins/Supplements	Nutritional supporting agents	Multivitamin, vitamin E
Other		Intravenous contrast dye, pneumococcal vaccine
