# Supplementary material for: Evaluating the Medication Regimen Complexity Score as a Predictor of Clinical Outcomes in the Critically Ill
Source: J Clin Med. 2022 Aug 11;11(16):4705. doi: 10.3390/jcm11164705 (PMC9410153; doi:10.3390/jcm11164705)
Supplement: Supplementary file 1 [file jcm-11-04705-s001.zip › Table S3.pdf]

Table S3: Model specifications of logistic regression and prediction models for intensive care unit mortality, length of stay (LOS), and mechanical ventilation (MV).

<table>
<tr><th>Model setup</th><th>Input variables</th><th>Output of interests</th></tr>
<tr><td colspan="2">Forward selection models</td><td rowspan="13">1. Inpatient mortality
0: survived and discharge
1: expired

2. Length of stay
0: <48 hours,
1: >=48 hours

3. Need for mechanical ventilation
0: no MV
1: on MV</td></tr>
<tr><td>Model I</td><td>Demographics*, APACHE II, SAPS II, CCI</td></tr>
<tr><td>Model II</td><td>Demographics, MRCI_24hours, MRCI_48hours, CCI</td></tr>
<tr><td>Model III</td><td>Demographics, MRCICU_24hours, MRCICU_48hours, CCI</td></tr>
<tr><td>Model IV</td><td>Demographics, APACHE II, SAPS II, CCI, MRCI_24hours, MRCI_48hours, MRCICU_24hours, MRCICU_48hours, and medication classes †</td></tr>
<tr><td colspan="2">Prediction models</td></tr>
<tr><td>Admission Model</td><td>Demographics, CCI, and SAPS II</td></tr>
<tr><td>MRCI Model</td><td>Demographics, MRCI, CCI</td></tr>
<tr><td>MRCI & SAPS II Model</td><td>Demographics, MRCI_24hours, MRCI_48hours, CCI and SAPS II</td></tr>
<tr><td>MRC-ICU Model</td><td>Demographics, MRC-ICU_24 hours, MRC-ICU_48hours, and CCI</td></tr>
<tr><td>MRC-ICU & SAPS II Model</td><td>Demographics, MRCICU_24 hours, MRCICU_48hours, CCI, and SAPSII</td></tr>
<tr><td>Medication Model</td><td>Demographics, medication classes</td></tr>
<tr><td>Full Model</td><td>Demographics, SAPS II, MRCI_24hours, MRCI_48hours, MRCICU_24 hours, MRCiCU_48hours, CCI, medication classes</td><td></td></tr>
</table>

*Demographics: Age, Gender, Height, Weight, BMI, and race;

†Medication classes (IV fluid/electrolyte/total parenteral nutrition, anti-infectives, analgesics and sedatives, cardiovascular, pulmonary, hematologic/anticoagulants, gastrointestinal, vasopressors, paralytics, psychiatric, endocrine, diuretics, genitourinary, vitamins/supplements, and others);

Abbreviations: SAPSII: Simplified Acute Physiology Score, CCI: Charlson comorbidity index, MRCI: medication regimen complexity index, MRCICU: medication regimen complexity index – intensive care unit.
