# Supplementary material for: Evaluating the Medication Regimen Complexity Score as a Predictor of Clinical Outcomes in the Critically Ill
Source: J Clin Med. 2022 Aug 11;11(16):4705. doi: 10.3390/jcm11164705 (PMC9410153; doi:10.3390/jcm11164705)
Supplement: Supplementary file 1 [file jcm-11-04705-s001.zip › Table S4.pdf]

Table S4. Comparison between patient characteristics and Medication Regimen Complexity Index (MRCI) scoring cohorts.

Characteristics	Low MRCI (n =157)	High MRCI (n =160)	*p*-value
**Demographics**			
Age, median (IQR), y	61.1 (48-76)	62.8 (52-75)	0.066
Sex, No. (%)			
Male	83 (53)	92 (57)	0.474
Race, No. (%)			
White	96 (61)	109 (68)	0.237
Black	12 (8)	14 (9)	0.877
Hispanic	18 (11)	19 (12)	> 0.999
Asian	3 (2)	1 (1)	0.601
BMI, median (IQR)	28.3 (23-31)	29.6 (23-33.2)	0.597
**Vital Signs**			
**Systolic Blood Pressure (mm Hg)**	**122.2 (108.3-132)**	**116.2 (102.4-128.7)**	**0.015**
**Diastolic Blood Pressure (mm Hg)**	**71 (61.4-80.2)**	**68.8 (59.8-78.5)**	**0.001**
**Mean Arterial Pressure (mm Hg)**	**88.3 (77.4-96.2)**	**84.4 (73.8-94.5)**	**0.002**
Heart Rate (beats/min)	88.2 (76-100.2)	94.2 (80-105)	0.956
Respiratory Rate (breaths/min)	19.9 (16.2-22.2)	21.2 (17.4-24.6)	0.203
Temperature (C°)	98.1 (97.5-98.6)	98.2 (97.5-99)	0.234
SaO_2 (mm Hg)	96.2 (95.1-98.6)	95.8 (94.2-98)	0.459
**Serum Laboratory Values**			
Sodium (mEq/L)	136.7 (134-139)	136.7 (133.8-139)	0.729
Potassium (mEq/L)	4 (3.6-4.3)	4.1 (3.6-4.5)	0.311
Chloride (mg/dL)	104.5 (101-108)	102.8 (100-107.7)	0.48
Carbon Dioxide (mEq/L)	23.8 (21.9-26.2)	22.3 (18-26)	0.104

Blood Urea Nitrogen (mg/dL)	26.6 (12-30.6)	30.1 (13-39.2)	0.238
Serum Creatinine (mg/dL)	1.5 (0.7-1.5)	1.6 (0.7-2)	0.726
Glucose (mg/dL)	159.9 (106.9-175.8)	198.8 (116.2-249.1)	0.438
Calcium (mg/dL)	8.3 (7.8-8.7)	8.1 (7.5-8.5)	0.954
Magnesium (mg/dL)	1.9 (1.7-2.1)	2 (1.6-2.2)	0.194
**Phosphate (mg/dL)**	**3.8 (2.9-4.2)**	**4.4 (2.8-4.7)**	**0.042**
WBC (x 10^3/mL)	11.5 (7.2-14)	12.5 (7.4-15.9)	0.214
Hemoglobin (g/dL)	10.4 (8.6-12.1)	9.8 (8.2-11.3)	0.329
Hematocrit (%)	32.7 (27.7-37.4)	30.8 (26-35.3)	0.498
Platelets (x 10^3/mL)	215.7 (142.7-274)	208.5 (138.2-268.5)	0.685
**Lactate (U/L)**	**3.9 (1.2-4.2)**	**5.9 (2.2-7.6)**	**0.043**
PT (seconds)	15.7 (11.7-15.7)	20.3 (12.8-24)	0.266
INR	1.5 (1.1-1.5)	2 (1.2-2.3)	0.293
**Albumin (g/L)**	**3.2 (2.8-3.6)**	**2.8 (2.4-3.2)**	**0.043**
Total_bilirubin (mg/dL)	1.6 (0.4-1.1)	1.8 (0.5-1.4)	0.255
Urine output every 6 hours (mL/hr)	57.5 (10.6-76.1)	61.6 (6.7-93.3)	0.191
eGFR (mL/min/1.73m^2)	69.1 (41-94)	64.8 (30.6-96)	0.875
**Duration(s)**			
Time on mechanical ventilation (hours)	43.1 (0-4)	78.3 (0-74)	0.222
ICU length of stay (hours)	89.5 (18-86)	133.5 (27-140.5)	0.396
**Scoring Assessment on ICU admission**			
**APACHE II**	**17.8 (13-21)**	**21 (15-26)**	**0.037**
SAPS II	12.9 (4-18)	19 (12-29)	0.149
**GCS at admission**	**12.8 (11-15)**	**10.7 (7-15)**	**0.029**
COVID-19 (positive)	26 (17)	26 (16)	> 0.999
**Comorbidities (ICD 10)**			
Hyperlipidemia (E78.5)	62 (39)	77 (48)	0.151

**Acute respiratory failure with hypoxia (J96.01)**	**50 (32)**	**75 (47)**	**0.009**
Hypertension (I10)	55 (35)	52 (32)	0.72
**Lactic acidosis (E87.2)**	**40 (25)**	**61 (38)**	**0.022**
Hypokalemia (E87.6)	43 (27)	59 (37)	0.092
Kidney Failure (N17.9)	46 (29)	50 (31)	0.798
**Hypo-osmolality + hyponatremia (E87.1)**	**33 (21)**	**56 (35)**	**0.008**
Was not resuscitated (Z66)	35 (22)	51 (32)	0.073
**Acute myocardial infarction (I21.A)**	**28 (18)**	**50 (31)**	**0.008**
**Unspecified sepsis (A41.9)**	**23 (15)**	**53 (33)**	**< 0.001**
