# Supplementary material for: Evaluating the Medication Regimen Complexity Score as a Predictor of Clinical Outcomes in the Critically Ill
Source: J Clin Med. 2022 Aug 11;11(16):4705. doi: 10.3390/jcm11164705 (PMC9410153; doi:10.3390/jcm11164705)
Supplement: Supplementary file 1 [file jcm-11-04705-s001.zip › Table S5.pdf]

Table S5. Comparison between patient characteristics and Medication Regimen Complexity Intensive Care Unit (MRC-ICU) score.

Measure	(Low) MRC-ICU (n=148)	(High) MRC-ICU (n=169)	*p*-values
**Demographics**			
Age, median (IQR), y	62 (51.8-77)	61.9 (50-72)	0.918
Sex, No. (%)			
Male	77 (52)	98 (58)	0.341
Race, No. (%)			
White	96 (65)	109 (64)	> 0.999
**Black**	**7 (5)**	**19 (11)**	**0.057**
Hispanic	16 (11)	21 (12)	0.786
Asian	3 (2)	1 (1)	0.524
BMI	28.6 (23-32)	29.2 (24-32)	0.413
**Vital Signs**			
Systolic Blood Pressure (mm Hg)	121.1 (104.6-134)	117.4 (103.7-125.8)	0.599
Diastolic Blood Pressure (mm Hg)	69.6 (60.3-78.4)	70.1 (60.9-79.5)	0.753
Mean Arterial Pressure (mm Hg)	86.8 (76-95.5)	85.9 (75.2-94.8)	0.983
Heart Rate (beats/min)	87.2 (76-98)	94.8 (80.1-106)	0.969
Respiratory Rate (breaths/min)	19.4 (16-21.4)	21.6 (17.3-25.2)	0.529
Temperature (C°)	98.1 (97.6-98.6)	98.1 (97.5-98.9)	0.548
SaO_2 (mm Hg)	96.2 (95.1-98.7)	95.9 (94-98)	0.635
**Serum Laboratory Values**			
Sodium (mEq/L)	136.4 (134-139)	136.9 (134-139.1)	0.411
Potassium (mEq/L)	4 (3.6-4.3)	4.2 (3.6-4.5)	0.435
Chloride (mg/dL)	104.3 (101-108)	103 (100-107)	0.572

Carbon Dioxide (mEq/L)	23.4 (21-26)	22.7 (18.9-26)	0.515
Blood Urea Nitrogen (mg/dL)	26.1 (12-30.2)	30.5 (13-40.2)	0.969
Serum Creatinine (mg/dL)	1.5 (0.7-1.7)	1.6 (0.8-1.8)	0.87
Glucose (mg/dL)	175.6 (111.5-193.2)	183.5 (114.2-228.2)	0.336
Calcium (mg/dL)	8.3 (7.8-8.8)	8.1 (7.5-8.5)	0.998
Magnesium (mg/dL)	1.9 (1.6-2.2)	2 (1.7-2.2)	0.8
Phosphate (mg/dL)	3.9 (2.9-4.3)	4.4 (3-4.8)	0.805
WBC (x 10^3/mL)	11.4 (7.3-14.5)	12.6 (7.3-15.9)	0.113
**Hemoglobin (g/dL)**	**10.5 (9-12)**	**9.7 (8-11.2)**	**0.047**
Hematocrit (%)	33 (28.3-37.2)	30.5 (25.6-34.8)	0.059
Platelets (x 10^3/mL)	219.8 (148-257.8)	204.7 (129-274)	0.314
Lactate (U/L)	4.7 (1.2-6.5)	5.3 (2.1-7.6)	0.632
PT (seconds)	15.2 (11.9-16.2)	20.8 (12.2-29.2)	0.578
INR	1.4 (1.2-1.6)	2.1 (1.2-2.9)	0.546
Albumin (g/L)	3.1 (2.8-3.6)	2.9 (2.4-3.3)	0.549
Total_bilirubin (mg/dL)	0.9 (0.4-1)	2.3 (0.6-1.3)	0.21
Urine output every 6 hours (mL/hr)	54.5 (7.3-76.5)	64.2 (9.8-100)	0.608
eGFR (mL/min/1.73m^2)	67.4 (38.5-93.8)	66.3 (36.5-97)	0.484
**Duration(s)**			
Time on mechanical ventilation (hours)	30.2 (0-0)	87.8 (0-80)	0.157
ICU length of stay (hours)	73.2 (18-64)	145.4 (33-160)	0.109
**Scoring Assessment upon ICU admission**			
APACHEII	18.3 (13-21)	20.4 (15-25)	0.749
SAPS II	12.1 (4.8-16.2)	19.4 (12-29)	0.567
GCS at admission	13.2 (13-15)	10.5 (7-15)	0.346
COVID-19 (positive)	19 (13)	33 (20)	0.146

**Comorbidities (ICD 10)**			
Hyperlipidemia (E78.5)	66 (45)	73 (43)	0.891
**Acute respiratory failure with hypoxia (J96.01)**	**42 (28)**	**83 (49)**	**< 0.001**
Hypertension (I10)	43 (29)	64 (38)	0.124
Lactic acidosis (E87.2)	39 (26)	62 (37)	0.064
Hypokalemia (E87.6)	40 (27)	62 (37)	0.086
Kidney Failure (N17.9)	46 (31)	50 (30)	0.868
**Hypo-osmolality hyponatremia (E87.1)**	**32 (22)**	**57 (34)**	**0.023**
**Was not resuscitated (Z66)**	**32 (22)**	**54 (32)**	**0.053**
**Acute myocardial infarction (I21.A)**	**27 (18)**	**51 (30)**	**0.02**
**Unspecified sepsis (A41.9)**	**21 (14)**	**55 (33)**	**< 0.001**
