# Supplementary material for: Evaluating the Medication Regimen Complexity Score as a Predictor of Clinical Outcomes in the Critically Ill
Source: J Clin Med. 2022 Aug 11;11(16):4705. doi: 10.3390/jcm11164705 (PMC9410153; doi:10.3390/jcm11164705)
Supplement: Supplementary file 1 [file jcm-11-04705-s001.zip › Table S6.pdf]

Table S6. Alternative method for variable selection. Four L1 penalization techniques (LASSO) for ICU mortality, ICU length of stay, and need for mechanical ventilation.

Selected features	Mortality<br>OR	Length of Stay<br>OR	Mechanical Ventilation<br>OR
**Model I**			
Age	1.01	1.01	-
BMI	-	1.01	-
Hispanic	4.21		1.01
SAPSII at admission	1.11	1.05	1.14
APACHE II at admission	1.12	-	-
CCI	-	1.11	-
**Model II**			
Age	1.02	1.01	-
BMI	-	1.01	-
Height	-	-	1.03
Hispanic	4.23	-	1.40
MRCI at 24hours	1.01	1.01	1.01
MRCI at 48hours	1.01	-	1.01
CCI	-	1.11	-
**Model III**			

Age	1.02	1.01	-
BMI	-	1.00	-
Height	-	-	1.07
Hispanic	4.32	1.12	1.59
MRC-ICU at_24hours	1.10	1.15	1.16
MRC-ICU at_48hours	1.07	-	1.10
CCI	-	1.11	-
**Model IV**			
Height	-	-	1.04
Hispanic	2.69	-	-
White	-	-	1.03
SAPSII_admission	1.05	-	1.12
APACHEII_admission	1.11	-	1.03
MRC-ICU at 24hours	-	1.04	1.15
Analgesics_Sedatives	-	1.26	4.52
Anti-Infectives	-	1.68	-
Cardiovascular_agents	-	1.69	-
Diuretics	1.02	2.63	1.64
Endocrine	-	1.10	-
Gastrointestinal agents	-	-	1.20

Anticoagulants	-	1.59	1.34
Paralytic_agents	1.69	2.63	20.75
Psychiatric_agents	-	1.43	1.64
Pulmonary_agents	1.17	1.20	1.94
Vasopressors	3.70	2.39	4.21
Vitamins	-	1.22	-
Others	1.42	-	-

The final models of logistic regression are reported using odds ratio (OR) of risk factors for logistic regression. If the variable was not selected , the cell marked with “-” .
