# Supplementary figures and images for: Evaluating the Medication Regimen Complexity Score as a Predictor of Clinical Outcomes in the Critically Ill
Source: J Clin Med. 2022 Aug 11;11(16):4705. doi: 10.3390/jcm11164705 (PMC9410153; doi:10.3390/jcm11164705)

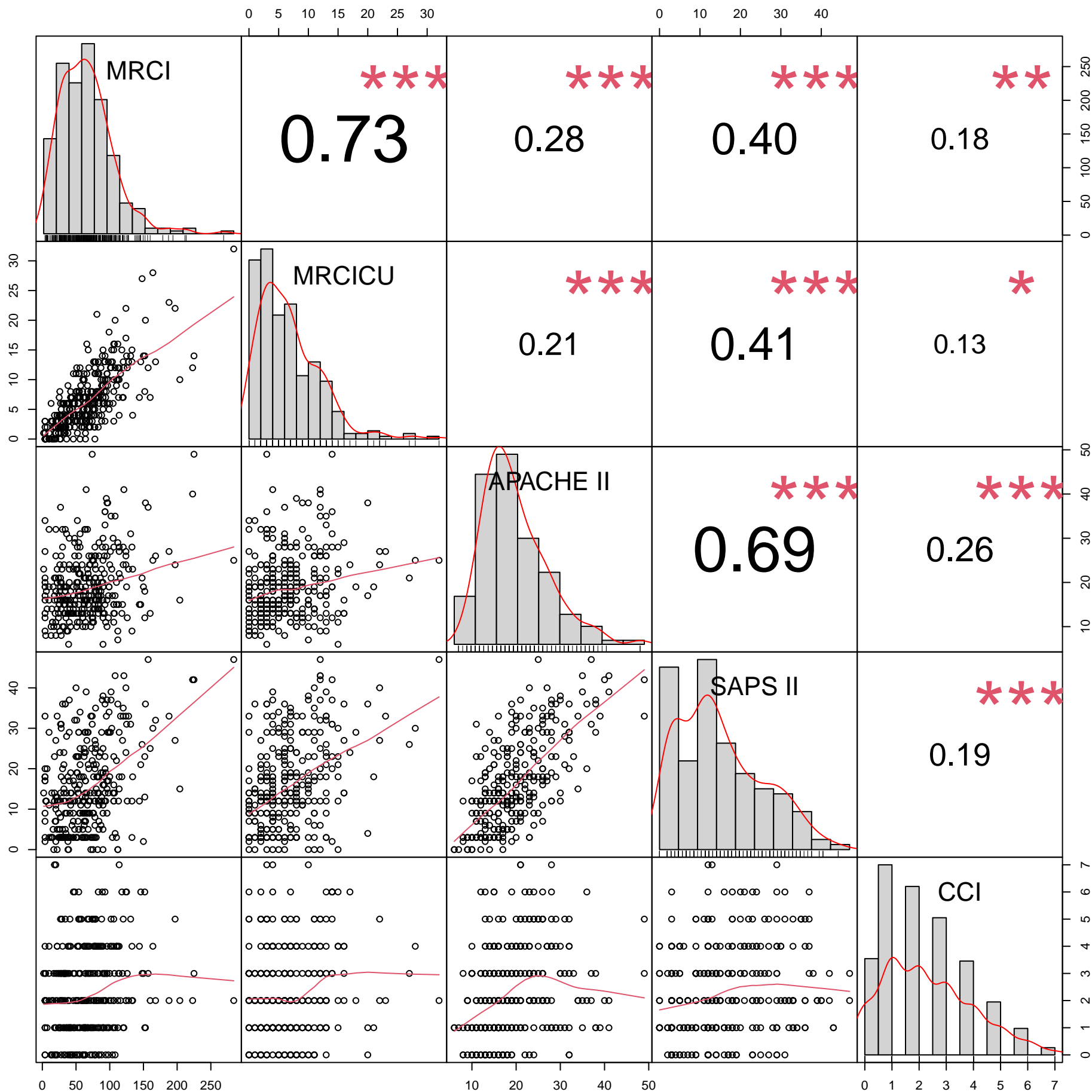

Supplement: Supplementary file 1 [file jcm-11-04705-s001.zip › Supp FigS1.pdf]

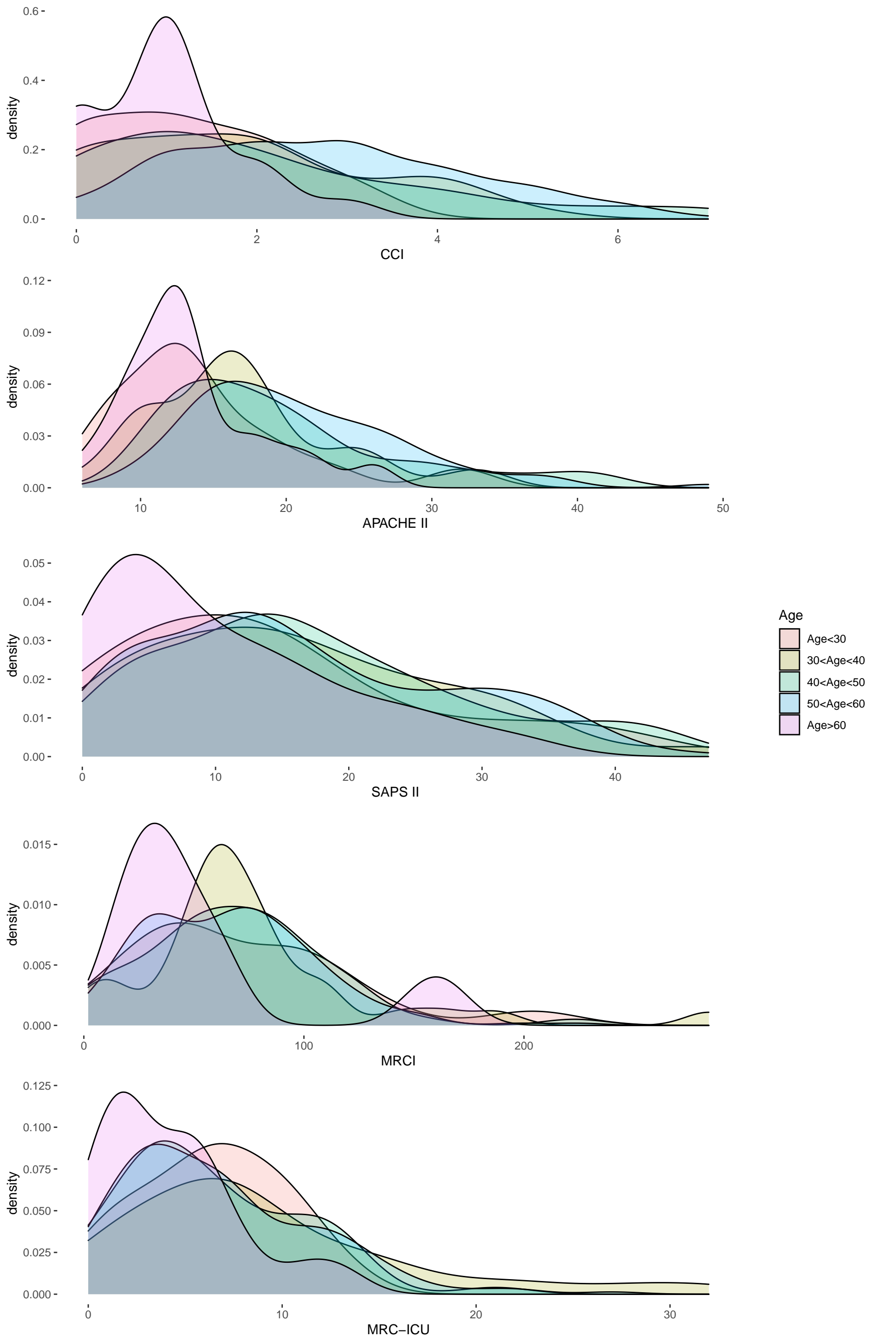

Supplement: Supplementary file 1 [file jcm-11-04705-s001.zip › Supp FigS2.pdf]
